# Cases of Bloody Infiltrations in the Labia Pudendi during, or Very Quickly after, Delivery; with Observations

**Published:** 1827-11

**Authors:** W. P. Dewees

**Affiliations:** Professor of Midwifery, Pennsylvania.


### MIDWIFERY.

*Cases of Bloody Infiltrations in the Labia Pudendi during, or very quickly after, Delivery; with Observations.* By W. P. Dewees, M.D. Professor of Midwifery, Pennsylvania. (Condensed from the *Philadelphia Journal.)*

During labour a variety of accidents may occur to the parts concerned in this operation, among which the one about to be noticed is not the least formidable in appearance, nor the least tedious in the cure. The disease to which I allude is the sudden and excessive distention of the labia pudendi, or only one of them, with blood, from some neighbouring vessel having given way, either during the progress, or very quickly after, the delivery of the child; or in some cases immediately after the expulsion of the head.

This complaint is generally confined to one labium: I have never seen it otherwise, though cases are related where it has happened to both. Thus Baudelocque mentions a case, on the authority of Solayres, where the labia were equally affected. This is certainly not usual; and perhaps may be accounted for from the peculiar nature of its causes,—namely, a varicose condition of the veins of the labia and vagina.

This accident, in every instance in which I have witnessed it, has taken place after the delivery of the child, though not always immediately after; but this is by no means constant, as we are informed by Drs. Maitland and Perfect, that the swelling occurred to them before the child was delivered. Dr. Maitland says, in his patient he found a soft tumor covering the os externum, very much resembling the distended membranes, which proved to be the right labium pudendi distended to the enormous size of a child's head.

Mr. Burns is of opinion that this swelling is owing to the rupture of a vessel within the nympha; but it is hardly probable that any vessel belonging to these parts would yield so suddenly such an enormous quantity of blood as is sometimes expended; for as much as five pounds have been discharged: in this case the patient died. I am of opinion that the blood proceeds from vessels situated rather within the vagina; and those which compose the vaginal plexus, immediately behind the corpus spongiosum, are those most likely to suffer during the passage of the child's head, and to furnish this large quantity of blood.

This complaint has been mistaken for the distended and protruding membranes, and for an hernia; but a careful examination of the deranged part will soon remove these errors: for it exhibits neither the position nor the colour presented in either of the cases with which it has been confounded. Its position is lateral, unless both labia are involved; in which case the natural sulcus must be observable; and its colour is that of extreme lividity, or entirely black, which resembles neither the membranes nor hernia.

The internal lining of the labium gives way sometimes from the excessive distention it has been made to suffer: this permits a quantity of fluid blood or coagula to escape, which tends very much to diminish the extreme anguish of the patient. In all instances of this kind much pain is endured, and in some cases it has been so severe as to cause syncope: a case of this kind is related by Dr. Reeve in the ninth volume of the London Medical Journal. Sometimes the tumor bursts before the child is born: Dr. Perfect relates a case of this kind; and the first case related below may be considered as a similar instance.

But, if this bursting does not take place, as sometimes happens when the size of the tumor is not enormous, the internal face of the labium is sure to yield in a short time, from gangrene taking place through its whole extent. This condition has been preceded, in two of the cases I have witnessed, by innumerable vesications, containing a yellowish serum, spreading themselves over the whole surface of the tumor formed by the stretching of the internal membrane of this part, but which, very soon after the swelling has acquired considerable size, yields, from the loss of life; and the patient, in consequence, feels considerable relief.

When the part sloughs, it exposes a large surface of coagulated blood, which quickly becomes decomposed, and yields a stench that is altogether intolerable.

Should the parts not give way, the pain arising from distention is agonising; fever of a very active kind is quickly kindled; delirium sometimes attends, and the woman's life becomes threatened. Her sufferings are also augmented by the retention of urine, as its passage is prevented by the tumor pressing firmly against the meatus externus of the urethra. The patient can lie only upon her back, with her knees drawn up, and the thighs widely separated. She cannot bear the pressure of the bed-clothes, nor the lightest applications; therefore it is vain to offer relief, until the distended parts yield spontaneously, or are made to do so by artificial means.

When fever attends, blood-letting must be employed to an extent that will ensure the reduction of arterial action; and be repeated pro re nata. With a view to give the earliest opportunity for the extravasated blood to escape, a free incision should be made the whole length of the tumor with a scalpel, or the shoulder of a lancet. I am not certain whether this plan has ever been insisted on by any writer, when the tumor preserves its integrity; but, whether or no, I am convinced it is the best mode of treatment.

The bowels must be purged by any of the neutral salts; but the patient must not be permitted to rise during their operation. If she be permitted to get out of bed, it will create much unnecessary pain, besides incurring the risk of the renewal of the bleeding, by the rude and too sudden separation of a coagulum. The strictest antiphlogistic regimen should be observed. We are told of cases where the bleeding has been considerable after the part has given way; to arrest which the wound was crammed with lint, and the vagina itself firmly plugged. I have never seen any bleeding follow the plan just suggested; nor do I see how it can well occur, without attempts have been, too early and too rudely made to separate or remove the impacted coagula. This must be carefully avoided; and their separation confided strictly to the powers of the system, unless it be the portions that separate themselves in consequence of the putrefaction of the blood itself, or by very gentle pressure. The detached coagula, of course, should always be removed, as often as they may separate.

It contributes greatly to the comfort of the patient, as well as being important to the cure, to keep the parts as clean as possible by frequent washing: for this purpose plain soap and water is as useful as any other mere detergent. The charcoal poultice is highly important, and should be constantly employed. The wound may be washed with a mixture of the pyroligneous acid and water; and the same acid may be profitably employed in its concentrated form, by applying over the poultice folded linen wetted with it.

Before the wound heals, the patient generally becomes considerably weakened from the excessive discharge of pus, &c. Her strength should be supported by a decoction of bark, elixir of vitriol, and a more generous diet, provided no febrile irritation remains. The following cases will illustrate the routine of practice.

Case I.—April 24th, 1806, I was called to Mrs. G. who was in labour with twins. At one o'clock P.M. she was delivered of a female child. About ten minutes after its birth, the right labium pudendi became excessively swelled. The part was found, upon inspection, to be extended to its utmost bearing; extremely black; and nothing but the internal surface of the labium presented itself. Before I arrived, however, the tumor had burst, from the efforts made to expel the second child. When I examined the patient, there was little swelling remaining in the labium, but there was discovered a considerable opening from its superior portion to its insertion in the perineum. The second child was well situated; pelvis faulty; pains pretty frequent and severe; and great pain was experienced immediately above the pubes. In about fifteen minutes after my seeing the patient, the labium was again distended, and again it discharged itself: this took place four times before the birth of the child. This frequent bursting of the labium destroyed the connexion of the labia with each other so completely, as to leave nothing but the external skin at the perineum to support the pressure of the child's head when passing through the external parts: this proved insufficient to support the force with which it was urged against it, and an extensive laceration, even to the verge of the anus, took place, notwithstanding every precaution which a timely fear could suggest. She lost from the part by this laceration at least twelve ounces of blood.

25th.—Complains of no soreness in the parts; the swelling nearly subsided; is feverish; some slight after-pains; passes no water.

Ordered a teaspoonful of the sweet Spirit of Nitre, and a purgative enema.

26th.—She passed water freely after the remedies. From this time but little inconvenience was experienced, except that which arose from the lacerated perineum. She was confined for some time to a horizontal posture, and at the end of the month was pretty well recovered.

This case differs very much from the two about to be related: first, in the blood being discharged from the tumor almost as soon as formed; second, in the integrity of the perineum being very much injured by the repeated yielding of the labial tumor, and in a laceration being inevitable; third, in the wound healing up in the labia without trouble, in consequence of its cellular structure retaining no coagula.

Case II.—July 2d, 1809, Mrs. A. was delivered of her second child, which was very large, after a severe labour of four hours, at eleven o'clock A.M. She appeared very well after delivery, except the frequent recurrence of severe after-pains, which, however, were relieved by the use of opium. At nine o'clock P.M. she complained of much pain, soreness, and tension in the left labium pudendi, which, upon examination, was found to be very much swelled: it continued to increase until it acquired a very large size, and quickly became vesicated. The internal lining of the labium was stretched to extreme thinness, was very black, and studded all over with little blisters, which contained a yellowish serum. I made, with the point of a lancet, a number of punctures, from which issued a considerable quantity of bloody serum: this afforded much relief.

3d.—Pain rather less; fever and delirium; no discharge of urine, owing to the pressure of the tumor upon the mouth of the urethra. She was ordered to lose twelve ounces of blood; the urine was relieved by pressing the tumor to one side, and at the same time raising it a little. An incision was made with the shoulder of a lancet through the extent of the inner portion of the tumor: this brought into view the coagula with which the whole of the cellular structure of the part was completely engorged. Much relief followed this operation. A strict antiphlogistic regimen was ordered.

4th.—Pain and fever much diminished; urine relieved as yesterday; a considerable discharge of thin, grumous, fetid blood; bowels confined.

An ounce of the Sulphate of Magnesia was given; and the charcoal poultice was directed.

The wound was poulticed, and coagula occasionally removed by pressure till the 15th, when the coagulum was entirely evacuated. From this time the parts healed kindly, and in the sixth week they were entirely well. Her strength much improved by the bark in decoction, and the elixir of vitriol. I attended this lady with several children after this time, without the smallest accident happening to the parts.

Case III.—August 30th, 1809, Mrs. C. was delivered about five o'clock P.M. of a large first child, after a labour of six hours. At ten o'clock the same evening, I was sent for in great haste, in consequence of a large and sudden swelling taking place, soon after the midwife had taken her leave. Upon inspection, the left labium was found much distended, very livid, and extremely painful. The distention or tumor was not so great as in the preceding case: this, however, was perhaps in a degree owing to my having been sent for immediately after the part was observed to swell, and its further progress being interrupted by my puncturing the tumor in several places, which gave opportunity for a considerable quantity of the thinner part of the blood to escape, and afforded some relief, or at least prevented further distention. The part was ordered to be covered with a soft bread-and-milk poultice, and, as she was suffering much pain, a full dose of laudanum was directed.

31st.—Still in great pain; high fever; and the tumor as large as it was the preceding evening, and vesicated as in the former case. Directed her to be bled; made an incision the whole length of the tumor, which afforded much relief. The charcoal poultice was ordered. Matters remained pretty much the same until the

5th of September.—On my visit this day, I was enabled, without much force, to express a large portion of coagulum, and did so every succeeding day until the 15th. Considerable quantities had come away at every dressing; and at this time (the 15th) the sore was entirely free from it, and presented a large but a healthy surface. The wound was entirely closed by the end of the fifth week.

In neither of the three cases just related was there any fungus produced, to interrupt the progress of the cure; a circumstance much to the advantage of the patient.

The mode of treatment pursued in these cases appeared to succeed so well, that I have been induced to relate it pretty much in detail; and I have been more strongly induced to this, as I have met with no account of the particular mode of treating this accident, so painful and alarming to the patient, and embarrassing to a young practitioner.

